# Human Palatine Tonsils Are Linked to Alzheimer’s Disease through Function of Reservoir of Amyloid Beta Protein Associated with Bacterial Infection

**DOI:** 10.3390/cells11152285

**Published:** 2022-07-24

**Authors:** Jung Yeon Lim, Jung Eun Lee, Han Kyung Kim, Yeon-Joon Park, Jung Ho Jeon, Soon-A. Park, Naeun Lee, Il Hwan Lee, Do Hyun Kim, Seung Ho Yang, Jongman Yoo, Sung Won Kim

**Affiliations:** 1 Department of Otolaryngology-Head and Neck Surgery, Seoul St. Mary’s Hospital, College of Medicine, The Catholic University of Korea, Seoul 06591, Korea; jylim8921@gmail.com (J.Y.L.); qscw2002@naver.com (J.H.J.); ilhwanloves@hanmail.net (I.H.L.); dohyuni9292@naver.com (D.H.K.); 2 Department of Neurosurgery, St. Vincent Hospital, The Catholic University of Korea, Suwon 16247, Korea; eunree@nate.com (J.E.L.); 72ysh@catholic.ac.kr (S.H.Y.); 3 Department of Microbiology, CHA University School of Medicine, Seongnam 13415, Korea; kimhankyung123@gmail.com (H.K.K.); jongmanyoo@cha.ac.kr (J.Y.); 4 Department of Laboratory Medicine, Seoul St. Mary’s Hospital, College of Medicine, The Catholic University of Korea, Seoul 06591, Korea; yjpk@catholic.ac.kr; 5 Department of Neurosurgery, Seoul St. Mary’s Hospital, The Catholic University of Korea, Seoul 06591, Korea; bobby1127@naver.com; 6 Center for Integrative Rheumatoid Transcriptomics and Dynamics, The Catholic University of Korea, Seoul 06591, Korea; nelee2015@catholic.ac.kr

**Keywords:** amyloid beta, brain organoid, human palatine tonsil, *Staphylococcus aureus*, tonsil organoid

## Abstract

Amyloid-β (Aβ)-peptide production or deposition in the neuropathology of Alzheimer’s disease (AD) was shown to be caused by chronic inflammation that may be induced by infection, but the role of pathogenic-bacteria-related AD-associated Aβ is not yet clearly understood. In this study, we validated the hypothesis that there is a correlation between the Aβ-protein load and bacterial infection and that there are effects of bacteria, *Staphylococcus aureus* (*S. aureus*), on the Aβ load in the inflammatory environment of human tonsils. Here, we detected Aβ-peptide deposits in human tonsil tissue as well as tissue similar to tonsilloliths found in the olfactory cleft. Interestingly, we demonstrated for the first time the presence of *Staphylococcus aureus* (*S. aureus*) clustered around or embedded in the Aβ deposits. Notably, we showed that treatment with *S. aureus* upregulated the Aβ-protein load in cultures of human tonsil organoids and brain organoids, showing the new role of *S. aureus* in Aβ-protein aggregation. These findings suggest that a reservoir of Aβ and pathogenic bacteria may be a possible therapeutic target in human tonsils, supporting the treatment of antibiotics to prevent the deposition of Aβ peptides via the removal of pathogens in the intervention of AD pathogenesis.

## 1. Background

Alzheimer’s disease (AD) is a progressive neurodegenerative disorder characterized by pathological features such as extracellular amyloid plaques, intracellular neurofibrillary tangles, and neuronal loss in the brain [1,2]. The amyloid-β (Aβ) peptide is a major component of plaques in the brain of Alzheimer’s disease patients and is produced through the processing of the amyloid precursor protein (APP) by β- and γ-secretases; Aβ_1–40_ is the most abundant peptide, and Aβ_1–42_ is a major component of amyloid plaques [3,4,5,6]. However, the initiating factors or causes of AD are still unclear.

Importantly, Balin et al. noted that *Chlamydia* *pneumoniae*, an intracellular Gram-negative bacterium, was present in the post mortem AD brain [7,8]. The systemic infection by this pathogen was associated with an increase in the incidence of AD, and anti-*C. pneumonia* antibody titers were increased in the blood of many AD patients. *C. pneumonia* may also enter the brain directly through the olfactory system [9]. Viable bacteria were found around the plaques in the brains of AD patients [10]. Moreover, amyloid deposits were observed after the intranasal infection of mice with *C. pneumoniae* [11]_,_ suggesting that bacterial antigens triggered Aβ-amyloid production and extracellular deposition in the brain of mouse models [3].

A dominant reservoir of bacteria is increasingly being found in the body. *Staphylococcus aureus* (*S. aureus*) was recently shown to survive in Kupffer cells, and its reactivation can occur at any time under favorable conditions [12]. *S. aureus*, a Gram-positive pathogen, causes a variety of disease pathologies, ranging from relatively mild tissue lesions to severe invasive sepsis, pneumonia, and deep-tissue abscesses [13,14,15]. *S. aureus* is the causative agent of most hospital-acquired bacterial infections in developed countries [16,17]. Many clinical isolates of *S. aureus* possess a set of virulence factors that are capable of invasion and bloodstream dissemination even in the absence of major tissue trauma [18].

A brain abscess is a complication that can occur after surgery, a head injury, or improperly treated *S. aureus*-induced sepsis or meningitis [19]. Although meningitis is rarely considered a complication of *S. aureus* infection, an increasing number of clinical reports document meningitis due to *S. aureus* infection of unknown origin [20,21]. In these cases, the hematogenous spread of bacteria from the primary site of infection indicates that *S. aureus* can cross the blood–brain barrier and enter the central nervous system (CNS). Spitzer et al. demonstrated that amyloidogenic peptide Aβ_1–42_ bound to the surface of *S. aureus* in culture in vitro [22]. Interestingly, as observed with immunocytochemistry, scanning electron microscopy, and Gram staining, aggregation was accelerated when Aβ_1–42_ was incubated with *S. aureus* [22], suggesting that Aβ_1–42_ agglutination was accelerated in the presence of microorganisms.

*S. aureus* is mostly detected in the intracellular and extracellular reservoirs of the tonsils [23]. Palatine tonsils are a pair of lymphoid organs associated with the upper respiratory tract where inhaled antigens first come into contact with host defense cells [24]. Tonsillar hyperplasia and recurrent tonsillitis are common chronic diseases that cause several complications, including nasal obstruction, snoring, auditory-tube dysfunction, recurrent sinusitis, otitis media, obstructive sleep apnea, facial growth, and behavioral developmental changes [25]. These complications require patients to frequently undergo tonsillectomy, one of the most commonly performed pediatric surgical procedures worldwide [26].

In this study, we investigated the correlation between the AD-associated Aβ-protein deposition and pathogenic bacteria in human palatine tonsils and the effect of a specific bacterium, *S. aureus*, on the Aβ deposits in the inflammatory environment of human tonsils removed from patients during tonsillectomy.

## 2. Methods

### 2.1. Patients

A series of children or adults undergoing tonsillectomy to treat tonsillar hypertrophy were enrolled in this study after obtaining the approval of the ethics committee and appropriate informed consent from the participants. The study procedure utilizing human palatine tissue was conducted in compliance with Institutional Review Board of Seoul St. Mary’s Hospital (KIRB-20200103-022), The Catholic University of Korea, and the Declaration of Helsinki.

### 2.2. Immunohistochemistry

The obtained human tonsillar tissue, olfactory tissue, or organoids were fixed with 4% (*w*/*v*) PFA and treated with paraffin- or frozen-embedded sections for staining with hematoxylin and eosin (H&E). For the immunohistochemistry of Aβ deposits in human palatine tonsils, human olfactory mucosal epithelium or organoids were fixed and embedded, snap-frozen in liquid nitrogen, and stored at −80 °C until use. The tissues were sectioned using a freezing microtome (Leica Camera, Wetzlar, Germany), pretreated with 97% formic acid, and incubated with the mouse anti-Aβ antibody (6E10, 1:100; BioLegend, San Diego, CA, USA, 803002) or anti-Aβ_42_ antibody (1:500; Novus Biologicals, Centennial, CO, USA; NBP2-44113) for 1 h at room temperature (RT). Subsequently, the sections were incubated with the biotinylated horse anti-mouse IgG antibody (1:200; Vector Laboratories, Burlingame, CA, USA) and FITC–streptavidin, or the tissues were incubated with the goat-rabbit Alexa Fluor 546 antibody (1:1000; Molecular Probes, Eugene, OR, USA; www.thermofisher.com, 1 December 2021). To determine whether Aβ staining was specific, an immunizing-peptide-blocking experiment was performed. Before the staining of the tissue sections, the Aβ_42_ antibody (1:300; Novus Biologicals) was neutralized by incubation with human APP synthetic peptide (Invitrogen, Carlsbad, CA, USA; PEP-0851) or human Aβ_1–42_ peptide (Alpha Diagnostic Intl. Inc. San Antonio, TX, USA; BAM422-P) for 1 h at RT. Subsequently, the sections were incubated overnight at 4 °C with primary anti-Aβ_42_ or neutralized antibodies and incubated with goat anti-rabbit Alexa Fluor 546 antibody (1:1000; Molecular Probes). For the immunofluorescence of *S. aureus* or E-cadherin, the tissue sections were incubated overnight at 4 °C with primary anti-*S. aureus* (1:500; Abcam, Cambridge, UK; ab2090) or anti-E-cadherin (1:500; Santa Cruz Biotechnology, Inc., Dallas, TX, USA; SC-8426) antibodies and incubated with the goat anti-rabbit Alexa Fluor 546 antibody. The nuclei were labeled with DAPI (1:1000; Sigma-Aldrich), and cell fluorescence was observed using a Zeiss LSM510 confocal microscope (Carl Zeiss, Jena, Germany).

### 2.3. Western Blots

For the Western blots of Aβ, wild-type (WT) mice and transgenic (Tg) mice expressing five mutants of human AβPP and PS1 (5 × FAD) (16 weeks of age; male; The Jackson Laboratory, Bar Harbor, ME, USA) were used in accordance with the institutional guidelines under conditions approved by Institutional Animal Care and Use Committee of The Catholic University of Korea. Human tonsillar tissues and mouse brain tissues were homogenized and sonicated in RIPA buffer (Thermo Fisher Scientific, Waltham, MA, USA) supplemented with 8M urea containing protease inhibitors (GenDEPOT, Inc., Barker, TX, USA). The supernatant was separated from the homogenates using centrifugation at 20,000× g for 20 min at 4 °C. For the Western-blot analyses of Aβ, protein samples were loaded onto NuPAGE 15% (*w*/*v*) Bis-Tris Gels (Thermo Fisher Scientific, Waltham, MA, USA) and transferred to a nitrocellulose membrane (0.11 µm pores; Whatman, GE Healthcare). The membrane was blocked with 5% (*w*/*v*) milk and incubated with primary antibodies against Aβ (6 × 10^10^; 1:100; BioLegend; 803002), *S. aureus* (1:500; Abcam; ab2090), and β-actin (1:1000; Santa Cruz Biotechnology; SC47778) and incubated with horseradish-peroxidase-conjugated secondary antibodies. The membrane was developed using enhanced chemiluminescence detection reagents (Thermo Fisher Scientific, Waltham, MA, USA).

### 2.4. Organoid Formation from Human Tonsillar Tissue

Human tonsil organoids were generated from human tonsillar tissue as previously described [27]. In brief, tonsils were obtained from patients via tonsillectomy. The samples were chopped and washed with D-PBS (LB001-02; Welgene, Daegu, Korea) and then enzymatically digested with advanced DMEM/F12 (11330-032; Gibco, Grand Island, NY, USA) containing 1 mg/mL collagenase II (17101015; Gibco) for 2 h at 37 °C. After digestion, isolated cells were embedded in Matrigel (354230; Corning, Inc., Corning, NY, USA), seeded in a 48-well plate (SPL, Inc., Gyeonggido, Korea), and incubated with 5% CO_2_ at 37 °C for 10 min to polymerize the matrices. Tonsil organoids were cultured in advanced DMEM/F12 supplemented with antibiotic–antimycotic (Thermo Fisher Scientific, Fisher Scientific, Waltham, MA, USA), GlutaMAX (Thermo Fisher Scientific), B27 (Invitrogen, Carlsbad, CA, USA), 10% R-spondin1-conditioned media and the following growth factors: 50 ng/mL recombinant murine HGF (315-23; PeproTech, Rocky Hill, NJ, USA), 100 ng/mL noggin (cyt-600; ProSpec, St. Paul, MN, USA), 20 nM A83-01 (SML0788; Sigma, St. Louis, MO, USA), 50 ng/mL human FGF10 (ATGP1387; ATGen, Seongnam, Korea), 20 ng/mL human bFGF (100-18B; Peprotech), 10 μM prostaglandin E2 (3632464; BioGems, Westlake Village, CA, USA), and 10 mM nicotinamide (N0636; Sigma). Neuregulin1 (5 nM; 100-03; Peprotech) was added only to the nasal-cavity-mucosa-derived tonsil-organoid cultures. After the cells were passaged, 10 μM Y-27632 (1254; Tocris Biosciences, Bristol, UK) was added to the culture medium for 2 days.

### 2.5. Treatment of Tonsil Organoids with S. aureus

In this study, an *S. aureus* clinical isolate obtained from a patient with tonsillectomy was used as treatment for tonsillar hypertrophy. The *S. aureus* inoculum was prepared by suspending an *S. aureus* colony in TSB and incubating it at 37 °C for 18 h. The bacterial suspension was centrifuged and washed with PBS; its optical density was adjusted to 1 × 10^8^ CFU/mL of *S. aureus*. Human-tonsil-organoid culture and *S. aureus* treatment were performed as follows: (i) The human tonsil organoids were cultured in a medium. (ii) *S. aureus* diluted in advanced DMEM/F-12 (without FBS and antibiotics) and tonsil organoids mixed with Matrigel in a 1:1 ratio were plated on a 48-well culture plate at a multiplicity of infection (MOI) of 10:1 (*S. aureus* to organoid culture). (iii) Tonsil organoids and *S. aureus* were next cultured together at 37°C in a 5% CO_2_ humidified incubator for 4 days. (iv) The tonsil organoids were plated to remove the bacteria in Matrigel, and a cell culture with tonsil growth medium supplemented with penicillin/streptomycin (antibiotics; Invitrogen, Carlsbad, CA, USA) was performed. (v) After 2 days, tonsil organoids were harvested and fixed for immunofluorescence analyses. 

### 2.6. Human Induced Pluripotent Stem Cell (iPSC) Culture

The CMC-hiPSC-011 cell line was used for all experiments. The study procedure utilizing CMC-hiPSC-011 was conducted in compliance with Institutional Review Board of Seoul St. Mary’s Hospital (KIRB-2019127-001), The Catholic University of Korea, as well as informed-consent regulations and the Declaration of Helsinki. The CMC-hiPSC-011 cell line was previously described [28] and was a kind gift from Dr. Joo (The Catholic University, Korea). hiPSCs were cultured using mTeSR1 medium (Stem Cell Technologies, Cambridge, MA, USA; 85850) under feeder-free culture conditions. Subcultures were performed enzymatically using Accutase (Thermo Fisher Scientific, Waltham, MA, USA; A1110501) by splitting colonies in clumps every 6–7 days, followed by replating on vitronectin-coated dishes.

### 2.7. Generation of Human Brain Organoids and Treatment with S. aureus

Organoids were generated using a STEMdiff Cerebral Organoid Kit (Stem Cell Technologies; 08570) assay following the manufacturer’s instructions. CMC-hiPSC-011 at 90% confluence was dissociated into single cells using Accutase (5 min, 37 °C) and resuspended in embryoid body (EB) formation medium with 10 μM Y27632 (Sigma-Aldrich Co., St. Louis, MO, USA; Y503), an ROCK inhibitor, and diluted to a concentration of 9 × 10^3^ cells per mL. Then, 100 μL of cell suspension was seeded in a low-attachment 96-well U-bottom plate (Corning) to form single EBs. The medium was replaced with induction medium every 2–3 days and then maturation medium. Human-brain-organoid culture and *S. aureus* treatment were performed as follows: (i) The human brain organoids were cultured in maturation medium. (ii) *S. aureus* was suspended in maturation medium and then added to the brain-organoid culture at an MOI of 10:1 (*S. aureus* to organoid culture) at 37 °C in a 5% CO_2_ humidified incubator for 2 h or 18 h. (iii) The brain organoids were washed twice with PBS to remove any nonadherent bacteria; then, fresh maturation medium was added, and the brain organoids were harvested and fixed for immunofluorescence analyses. Moreover, 1.0 × 10^6^ CFU/mL *S. pyogenes* (ATCC, Manassas, VA, USA; *Streptococcus pyogenes* Rosenbach) was used as treatment for the human brain organoids for 5 h. The expression of Nestin (1:500; Santa Cruz Biotechnology Inc., Dallas, Texas, USA; SC-23927), β-III tubulin (1:500; BioLegend, San Diego, CA, USA; 801201), and Iba-1 (1:500; Wako, Osaka, Japan) in the brain organoids was observed using a Zeiss LSM510 confocal microscope (Carl Zeiss).

### 2.8. Statistical and Reproducibility

All data from this experiment were expressed as the means (SD) from at least 3 independent experiments. Tukey’s post hoc ANOVA tests were used to determine whether group differences were statistically significant in multiple-comparison tests. Statistical differences between two different samples were determined with Student’s *t*-tests. In the statistical analyses, probability values < 0.05 were considered significant. In brain-organoid experiments for the quantification of Aβ-positive cells, cells were counted in 4 randomly selected nonoverlapping regions per section (four organoids per group). Stained-cell counts were analyzed using Image-Pro Plus software (Media Cybernetics, Inc., Rockville, MD, USA; http://www.mediacy.com (accessed on 1 December 2021).

## 3. Results

### 3.1. Aβ-Protein Deposition in Human Palatine-Tonsil Tissue

**Figure 1:**
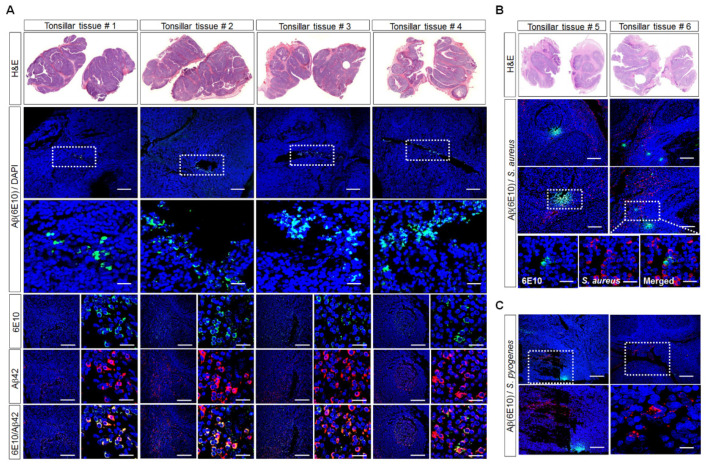
Microscopic images of human palatine-tonsil specimens subjected to immunohistostaining. (**A**) H&E images after the staining of the paraffin-embedded sections or confocal-microscopy images after the staining of the paraffin-embedded sections with the 6E10 antibody (green) or Aβ_42_ antibody (red) to detect Aβ deposition. The boxed area indicates the 6E10- or Aβ_42_-positive cells in the human palatine-tonsil specimens (tissue #1, 7 years old; tissue #2, 49 years old; tissue #3, 52 years old; tissue #4, 53 years old). Nuclei were labeled with DAPI (blue). Scale bars: 200 μm, 100 μm, 20 μm. (**B**) H&E images after the staining of the paraffin-embedded sections or confocal-microscopy images after the staining of the sections with the 6E10antibody l (green) or *S. aureus* antibody (red). The boxed area indicates the 6E10- or *S. aureus*-positive cells in the human palatine-tonsil specimens (tissue #5, 59 years old; tissue #6, 50 years old). Nuclei were labeled with DAPI (blue). Scale bars: 200 μm, 100 μm, 10 μm. (**C**) Confocal-microscopy images after the staining of the sections with the 6 × 10^10^ antibody (green) or *S. pyogenes* antibody (red). The boxed area indicates the 6E10- or *S. aureus*-positive cells in the human palatine-tonsil specimen. Nuclei were labeled with DAPI (blue). Scale bars: 200 μm, 100 μm, 10 μm. All images are representative of two or three independent experiments.

We obtained human palatine-tonsil tissues from patients following tonsillectomy and investigated whether these contained Aβ deposits by staining with the Aβ-peptide antibody 6E10, which recognizes most forms of Aβ as well as APP, and the Aβ_42_ antibody. The H&E staining of the paraffin-embedded human palatine-tonsil samples showed stratified surface epithelium with a multilayer structure. Interestingly, immunofluorescence staining revealed Aβ deposits around the tonsillar crypts and lymph nodes in the palatine-tonsil tissue sections obtained from patients of different ages. Moreover, the immunostaining of the tissue sections with both anti-6E10and anti-Aβ_42_ antibodies showed that many cells were double positive for 6E10and Aβ_42_ (Figure 1A). Moreover, we investigated the presence of *S. aureus* in palatine-tonsil tissues with immunostaining. The immunofluorescence analyses of the tissue sections with both anti-*S. aureus* and anti-6E10antibodies showed that *S. aureus* was clustered around or embedded in the Aβ deposits, and some *S. aureus* and Aβ were co-localized in the tissues (Figure 1B). Next, we investigated the presence of *Streptococcus pyogenes* (*S. pyogenes)* in palatine-tonsil tissues with immunostaining. *S. pyogenes* is the most common cause of pharyngitis and tonsillitis, and in people with recurrent tonsillitis, the tonsils become enlarged. Most excised tonsils harbor intracellular *S. pyogenes*, indicating that the mucosal-associated lymphoid tissue is an important reservoir of bacteria [29]. *S. pyogenes* was present in palatine-tonsil tissues, but it was not clustered around or embedded in the Aβ deposits (Figure 1C).

### 3.2. Detection of Aβ Deposition and S. aureus in Human Palatine-Tonsil Tissue

**Figure 2:**
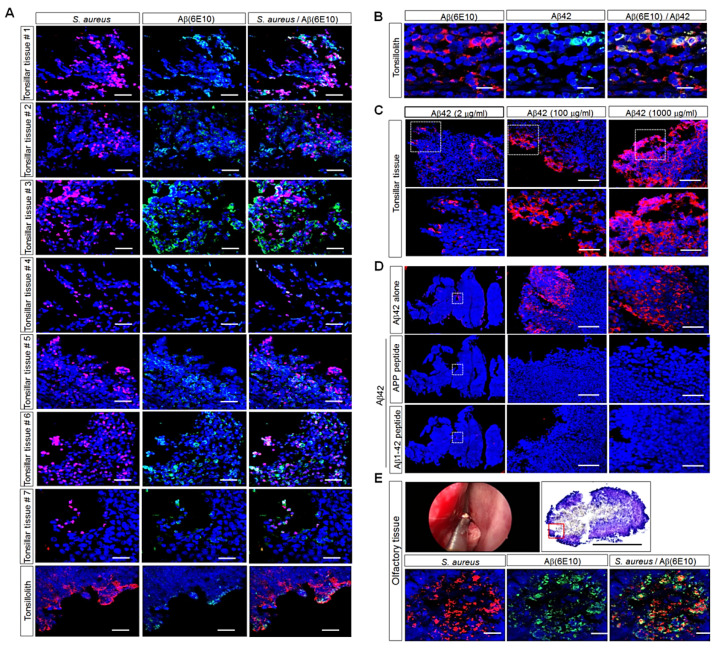
Histological analyses of human palatine-tonsil specimens or human olfactory mucosal epithelium subjected to immunohistostaining. (**A**) Confocal-microscopy images after the double staining of OCT-embedded human palatine-tonsil sections with antibodies against *S. aureus* (red) and Aβ-peptide-specific 6E10 (green) (tissue #1, 9 years old; tissue #2, 11 years old; tissue #3, 16 years old; tissue #4, 43 years old; tissue #5, 7 years old; tissue #6, 8 years old; tissue #7, 8 years old). Nuclei were labeled with DAPI (blue). Scale bar: 20 μm. All images are representative of two or three independent experiments. (**B**) Confocal-microscopy images after the double staining of OCT-embedded human palatine-tonsil sections with antibodies against 6E10 antibody (green) or the Aβ_42_ antibody (red) to detect Aβ deposition. Scale bars: 20 μm, 10 μm. (**C**) Confocal-microscopy images after the staining of OCT-embedded human palatine-tonsil sections with three different concentrations of the anti-Aβ_42_ antibody (red) in to detect Aβ deposition. Scale bars: 50 μm, 20 μm. (**D**) Confocal-microscopy images after the staining of OCT-embedded human palatine-tonsil sections with anti-Aβ_42_ or neutralized antibodies (red) to detect Aβ deposition. Scale bars: 100 μm, 50 μm. (**E**) Confocal-microscopy images after the double staining of an OCT-embedded section similar to tonsilloliths found in the human olfactory mucosal epithelium with antibodies against *S. aureus* (red) and Aβ-peptide-specific 6E10 (green) (olfactory tissue, 80 years old). Nuclei were labeled with DAPI (blue). Scale bars: 100 μm, 20 μm. All images are representative of two or three independent experiments.

The most common bacterial isolate from human tonsillar specimens was *S. aureus* [23]. Moreover, *S. aureus* was the most prevalent pathogenic bacterium in our culture data from 50 human palatine-tonsil tissues. After obtaining seven human palatine-tonsil tissues, we investigated the presence of *S. aureus* and Aβ deposits in these tissues with immunostaining. The immunofluorescence analyses showed that *S. aureus* was present in seven patients and ranged from very occasional colonies and small localized groups of colonies to substantial clusters of bacteria (Figure 2A), suggesting that the levels of *S. aure*us and Aβ deposits were different in the seven individuals. Interestingly, immunofluorescence staining demonstrated that *S. aureus* was clustered around or embedded in the Aβ deposits and that some *S. aureus* and Aβ were co-localized in the seven different tissues. Moreover, tissue from a case of tonsillolith was positive for *S. aureus* and Aβ deposition, although the expression levels were lower than those in human tonsillar tissues (Figure 2A). The immunostaining of the tissue sections with both anti-6E10and anti-Aβ_42_ antibodies showed that many cells were double positive for 6E10and Aβ_42_ (Figure 2B). To determine whether Aβ-antibody staining was specific, we performed immunofluorescence staining with three different concentrations of the Aβ_42_ antibody or immunizing-peptide-blocking experiments in human palatine-tonsil tissue. The immunofluorescence analyses showed that the Aβ-protein level was greater in tonsil tissues incubated with anti-Aβ_42_ antibody at a concentration of 1000 μg/mL than in the tissues incubated with anti-Aβ_42_ antibody at a concentration of 2 μg/mL or 100 μg/mL (Figure 2C). The immunostaining of the tissue sections with the neutralized Aβ_42_ antibody (pre-incubated with the APP synthetic peptide or the Aβ_1–42_ peptide) abolished almost all of the fluorescence compared with the tissue sections incubated with the anti-Aβ_42_ antibody alone (Figure 2D). We next examined Aβ deposition and the expression of *S. aureus* in yellowish olfactory tissue, which was similar to the tonsilloliths found in the olfactory cleft, a very narrow space, in a mixed state with sticky mucus and bacteria due to the rapidly decreasing mucus in the elderly; this sample was composed of olfactory epithelium between the superior turbinate and nasal septum and collected during endoscopic endonasal skull-base surgery. In Figure 2E, the patient who had olfactory tissue surgically removed was recently diagnosed with AD during a post-operative follow-up and was being treated. Notably, the confocal-microscopy images displayed Aβ deposits and clustered *S. aureus*, and some *S. aureus* and Aβ were co-localized in the tissues, as shown in Figure 2E, suggesting that human palatine-tonsil tissue may be a possible inducer of AD due to the storage of Aβ protein.

### 3.3. Presence of APP Fragments in Human Palatine-Tonsil Tissue

**Figure 3:**
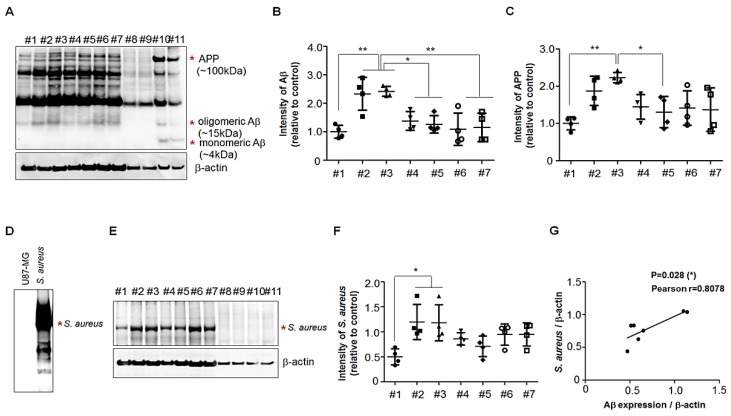
Expression of Aβ-specific 6E10 immunoreactive proteins in human palatine-tonsil specimens. (**A**) Western blots of SDS–PAGE gels of human palatine-tonsil tissue extracts using the primary anti-6E10antibody revealed multiple Aβ-specific bands in the human palatine tonsils (tissue #1, 9 years old; tissue #2, 11 years old; tissue #3, 16 years old; tissue #4, 43 years old; tissue #5, 7 years old; tissue #6, 8 years old; tissue #7, 8 years old; tissues #8–9, WT mice; tissues #10–11, 5 × FAD Tg mice). β-actin was used as a loading control. (**B**) Each bar represents the intensity of the Aβ-specific fragments of the ~15 kDa band from the Western blots. Values are the means (SD). A one-way ANOVA was used to determine whether group differences were significant in nonparametric multiple-comparison tests. ** *p* < 0.01, * *p* < 0.05. (**C**) Each bar represents the intensity of the APP-specific fragments of the ~100 kDa band from the Western blots. Values are the means (SD). A one-way ANOVA was used to determine whether group differences were significant in nonparametric multiple-comparison tests. ** *p* < 0.01, * *p* < 0.05. (**D**,**E**) Western blots of SDS–PAGE gels of extracellular protein fraction of human glioma cell line U-87MG or *S. aureus* and human palatine-tonsil tissue extracts. Immunodetection using the *S. aureus* antibody revealed multiple S. aureus-specific bands in the human palatine tonsils. (**F**) Each bar represents the intensity of the *S. aureus*-specific ~55 kDa band from the Western blots. Values are the means (SD). A one-way ANOVA was used to determine whether group differences were significant in nonparametric multiple-comparison tests. * *p* < 0.05. (**G**) There was a correlation between the levels of Aβ fragments and *S. aureus* in the-tonsil extracts.

The levels of two types of APP fragments, an ~15 kDa fragment and an ~55 kDa fragment, were elevated in the lumbar cerebrospinal fluid (CSF) of cognitively intact elderly people at risk for AD [30]. To investigate the presence of the APP fragment located N-terminally on Aβ in human palatine-tonsil tissue, we performed SDS-PAGE analyses of palatine-tonsil extracts (supplemented with 8 M urea) from seven patients and brain extracts from WT mice and 5 × FAD Tg mice and used the 6E10antibody for Western-blotting analyses. Multiple Aβ-specific bands were present in all palatine tonsils and 5 × FAD Tg mice brain, but not in WT mice, and we analyzed the levels of ~15 kDa in the samples (Figure 3A). There was a substantial difference in their intensities relative to that of the β-actin control among seven human palatine-tonsil tissues (Figure 3B). To further investigate whether the different levels of Aβ resulted from APP production in palatine tonsils, we analyzed the levels of the APP of the 100 kDa band from the Western blots (Figure 3A). There was a consistent change in the intensities, relative to the β-actin control (Figure 3C), of the levels of the Aβ fragments of ~15 kDa in the human palatine-tonsil samples. Therefore, it appeared likely that APP production regulated the Aβ levels in palatine-tonsil tissues. We next examined whether the levels of *S. aureus* were different in the extracts of seven human palatine-tonsil tissues. The Western blots of SDS-PAFE gels of tonsil extracts showed that multiple *S. aureus*-specific bands were present in all palatine tonsils (Figure 3D), but not in human glioma cell line U87-MG (Figure 3C), and we analyzed the levels of ~55 kDa in the samples. There was a consistent change in the intensities, relative to the β-actin control (Figure 3E), of the levels of Aβ fragments of ~15 kDa in the human palatine-tonsil samples. Furthermore, we observed a robust correlation between the levels of Aβ fragments and *S. aureus* in seven different tonsil specimens (Figure 3F).

### 3.4. Influence of S. aureus on Aβ-Protein Expression in Human Palatine-Tonsil-Tissue-Derived Tonsil Organoids

**Figure 4:**
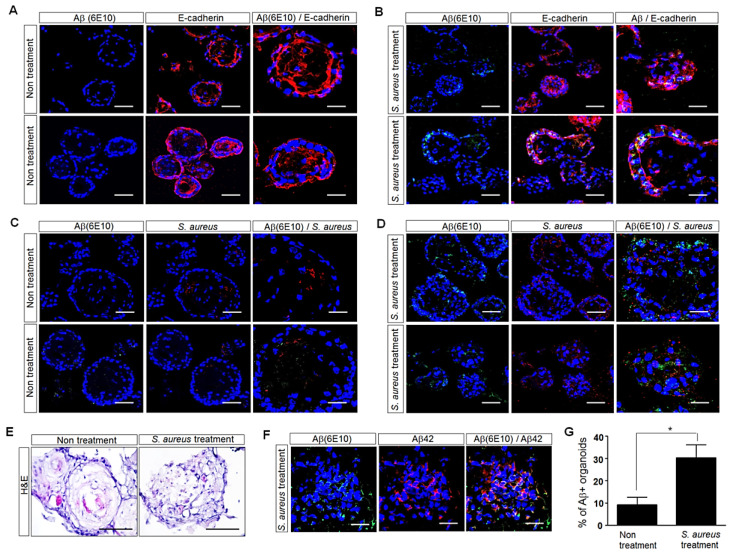
Histological analyses of human-palatine-tonsil-specimen-derived tonsil organoids subjected to immunohistostaining. (**A**,**B**) Confocal-microscopy images of human tonsil organoids cultured in the absence or presence of *S. aureus* at an MOI of 10 after the double staining of the OCT-embedded sections with the Aβ-specific 6E10antibody (green) and the antibody against tonsil-epithelium marker E-cadherin (red). Nuclei were labeled with DAPI (blue). Scale bars: 50 μm, 20 μm. All images are representative of two or three independent experiments. (**C**,**D**) Confocal-microscopy images of human tonsil organoids cultured in the absence or presence of *S. aureus* at an MOI of 10 after the double staining of the OCT-embedded sections with antibodies against Aβ-specific 6E10 (green) and *S. aureus* bacteria (red). Nuclei were labeled with DAPI (blue). Scale bars: 50 μm, 20 μm. All images are representative of two or three independent experiments. (**E**) H&E staining of the OCT-embedded sections 5 days after incubation of human tonsil organoids in culture medium. Scale bar: 100 μm. (**F**) Confocal-microscopy images after the double staining of OCT-embedded organoid sections with antibodies against 6E10antibody (green) or Aβ42 antibody (red) to detect Aβ deposition. Scale bar: 20 μm. (**G**) Aβ-positive organoids were counted. Each bar represents the mean percent of the Aβ-positive organoids. Values are the means (SD). A Student’s t-test was used to determine the statistical differences between two different samples. * *p* < 0.05.

To further investigate the possible impact of *S. aureus* on Aβ-protein levels in human palatine-tonsil tissue, we generated human tonsil organoids from this tissue and then added *S. aureus* for 5 days in the organoid culture. Five days after treatment with *S. aureus*, the morphology of the human tonsil organoids was observed with H&E staining. Treatment with *S. aureus* induced damage in the tonsil organoids compared with the tonsil organoids cultured in the absence of *S. aureus* (Figure 4E). The immunofluorescence analyses of E-cadherin showed the presence of a basal cell layer in the *S. aureus*-treated or untreated human tonsil organoids. Interestingly, the immunofluorescence analyses of 6E10visualized the Aβ protein, which was increased in the tonsil organoids cultured in the presence of *S. aureus* compared with the tonsil organoids cultured in the absence of *S. aureus* (Figure 4A,B,E). The immunostaining of the organoid sections with both anti-6E10and anti-Aβ_42_ antibodies showed that many cells were double positive for 6E10and Aβ_42_ (Figure 4F). The treatment of the tonsil organoids with *S. aureus* resulted in approximately 3.0-fold more Aβ-positive organoids than in untreated organoids (Figure 4G). Moreover, greater levels of *S. aureus* were detected around the Aβ aggregates in the human tonsil organoids cultured in the presence of *S. aureus* than in the tonsil organoids cultured in the absence of *S. aureus* (Figure 4C,D). These results demonstrated the increase in Aβ-protein levels in response to *S. aureus* in human tonsil organoids, suggesting that Aβ and *S. aureus* may interact in human palatine tonsils.

### 3.5. Influence of S. aureus on Aβ-Protein Expression in Human iPSC (hiPSC)-Derived Human Brain Organoids

**Figure 5:**
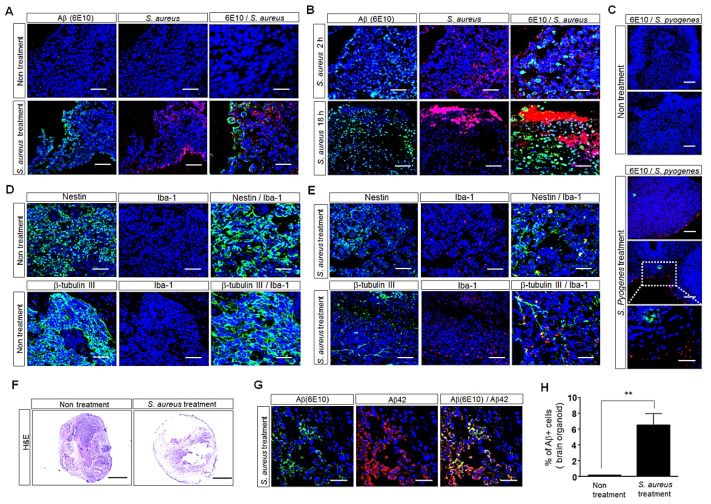
Histological analyses of hiPSC-derived human brain organoids subjected to immunohistostaining. (**A**) Confocal-microscopy images of human brain organoids cultured for 2 h in the absence or presence of *S. aureus* at an MOI of 10 after the double staining of the OCT-embedded sections with the Aβ-specific 6E10antibody (green) and the antibody against *S. aureus* (red). Nuclei were labeled with DAPI (blue). Scale bars: 50 μm, 20 μm. All images are representative of two independent experiments. (**B**) Confocal-microscopy images of human brain organoids cultured for 2 h or 18 h in the presence of *S. aureus* at an MOI of 10 after the double staining of the OCT-embedded organoid sections with the Aβ-specific 6E10antibody (green) and the antibody against *S. aureus* (red). Nuclei were labeled with DAPI (blue). Scale bars: 50 μm, 20 μm. (**C**) Confocal-microscopy images of human brain organoids cultured for 5 h in the presence of *S. pyogenes* after the double staining of the OCT-embedded organoid sections with the Aβ-specific 6E10antibody (green) and the antibody against *S. pyogenes* (red). Nuclei were labeled with DAPI (blue). Scale bars: 100 μm, 10 μm. (**D**,**E**) Confocal-microscopy images of human brain organoids cultured in the absence or presence of *S. aureus* at an MOI of 10 after the double staining of the OCT-embedded sections with antibodies against Nestin (green) and Iba-1 (red) or against β-tubulin III (green) and Iba-1 (red). Nuclei were labeled with DAPI (blue). Scale bars: 50 μm, 20 μm. All images are representative of two or three independent experiments. (**F**) H&E staining of the OCT-embedded sections at 2 h after incubation of the human brain organoids cultured in the absence or presence of *S. aureus* at an MOI of 10. Scale bar: 500 μm. (**G**) Confocal-microscopy images after the double staining of OCT-embedded organoid sections with antibodies against 6E10antibody (green) or Aβ_42_ antibody (red) to detect Aβ deposition. Scale bar: 20 μm. (**H**) Aβ-positive organoids were counted. Each bar represents the mean percent of the Aβ-positive cells in the organoids ± SD. Values are the means (SD). A Student’s t-test was used to determine the statistical differences between two different samples. ** *p* < 0.01.

To further validate the effect of *S. aureus* on Aβ levels, we generated cerebral organoids by culturing hiPSCs (Figure 5F) and then added *S. aureus* to the organoid cultures. After treatment with *S. aureus*, the morphology of the human brain organoids was observed with H&E staining. Staining showed that *S. aureus* treatment induced cell death in the brain organoids compared with the brain organoids cultured in the absence of *S. aureus* (Figure 5F). The immunofluorescence analyses of 6E10showed that the Aβ-protein level was greater in the brain organoids cultured for 2 h in the presence of *S. aureus* than in the brain organoids cultured in the absence of *S. aureus* (Figure 5A). Moreover, the Aβ-protein level was greater in the brain organoids cultured for 18 h in the presence of *S. aureus* than in the brain organoids cultured for 2 h in the presence of *S. aureus* (Figure 5B). Treatment with *S. aureus* resulted in approximately 40-fold more Aβ-positive cells in the treated brain organoids than in the untreated organoids (Figure 5H). However, the Aβ-protein level was slightly increased in the brain organoids cultured in the presence of *S. pyogenes* compared with the brain organoids cultured in the presence of *S. aureus* (Figure 5C). The immunostaining of the organoid sections with both anti-6E10and anti-Aβ_42_ antibodies showed that many cells were double positive for 6E10and Aβ42 (Figure 5G). The immunofluorescence analyses showed that treatment with *S. aureus* reduced the expression of neuronal cells but increased the expression of inflammatory microglial cells in the brain organoids compared with the brain organoids cultured in the absence of *S. aureus* (Figure 5D,E). These results showed that Aβ expression increased in the culture of human tonsil organoids with *S. aureus*, as shown in Figure 4. Taken together, these results suggest that the interaction between Aβ protein and *S. aureus* may increase Aβ-peptide production, which can lead to Aβ-related AD.

## 4. Discussion

AD is a neurodegenerative disorder mainly characterized by the abundance of Aβ peptides generated from the APP in the brain [31]. Aβ peptides exist in a variety of different forms, including soluble, membrane-associated, and intracellular species, which may play far more important roles in the development of dementia than the extracellular plaque molecules in the brain. Aβ peptides are produced in significant amounts not only in the brain but also outside the CNS in skeletal muscle, platelets, and vascular walls [32,33,34]. Other non-neural tissues that express the APP include the kidney, spleen, pancreas, liver, testis, aorta, heart, lung, intestines, skin, adrenal salivary glands, and thyroid glands [35,36,37]. These distinct reservoirs allow Aβ peptides to be exchanged actively and dynamically between the brain and periphery. Recent studies showed that blood-derived Aβ can be transported to the brain and contribute to the pathogenesis of AD in the brain of mouse models. Moreover, *Porphyromonas gingivalis* infection was shown to enhance peripheral Aβ transportation in cerebral endothelial cells and Aβ accumulation in the brain of mouse models [38,39]. Several studies identified blood-based biomarkers of AD pathology, such as plasma Aβ. A test for blood-based biomarkers would be valuable, because it would be a simple, safe, and minimally invasive method compared with brain positron emission tomography or magnetic-resonance-imaging analyses and cerebrospinal-fluid-biomarker analyses [40,41,42,43]. However, the lack of consistency in the results from blood-based biomarkers requires further validation and other feasible methods for the early and accurate diagnosis of AD. A recent study reported that elderly people with olfactory dysfunction were more than twice as likely to develop dementia five years later than those without olfactory dysfunction [44]. In APP/presenilin (PS1) transgenic mice, the deposition of Aβ began in the olfactory system and then spread to the brain [45]. Moreover, when an isotope-labeled Aβ peptide was injected into the ventricle of an experimental rat, it was observed that the Aβ peptide was transported from the brain to the nasal cavity through a nonhematogenous pathway [46]. Interestingly, Kim et al. demonstrated that the Aβ levels in nasal secretions was higher in AD patients than in individuals without cognitive impairment [47], suggesting that the detection of Aβ in nasal secretions may be a potential biomarker for predicting AD.

Tonsils are lymph glands at the back of the throat. These glands are an integral part of the body’s immune system and help to defend against invading microorganisms entering through the mouth or the nose [24]. A diverse range of microbes, including both commensal and pathogenic organisms, were isolated from human tonsils. Emerging evidence highlighted the association between the enlargement of the tonsils (tonsillar hyperplasia) and the microorganisms existing in these tissues [48,49,50,51,52]. Surgery is required because tonsillar hyperplasia causes conditions such as obstructive sleep apnea (OSA) or recurrent tonsillitis (RT) caused by repeated infections [48]. Tonsillectomy is one of the most common surgical procedures performed in children, and an increasing number of surgeries are now being performed to treat sleep-apnea-related disorders such as OSA [48]. Interestingly, increased levels of AD-related Aβ_1–42_ peptides and PS1 were found in plasma samples from children with OSA compared with those of healthy children [53]. However, their expression levels were decreased significantly after adenotonsillectomy in children with OSA.

In this study, we investigated the expression of Aβ in human palatine tonsils collected from patients following tonsillectomy. Immunofluorescence staining with the 6E10body, which is specific to Aβ peptides, revealed Aβ deposits around the tonsillar crypts and lymph nodes in the palatine-tonsil tissue sections obtained from patients of different ages (Figure 1). Moreover, Western blots using the 6E10antibody demonstrated the presence of APP fragments located N-terminally on Aβ in human palatine-tonsil tissue; more notably, there was a significant difference in the expression levels of a soluble fragment of ~15 kDa in palatine-tonsil extracts from seven patients (Figure 3). There is consensus that neurological dysfunction in AD is closely related to Aβ oligomers present in the human brain and biological fluids, suggesting that Aβ oligomers may serve as biomarkers for the clinical diagnosis of AD [54,55]. Recently, the levels of an APP fragment (a ~15 kDa fragment) were shown to be elevated in the lumbar CSF of cognitively intact elderly people at risk for AD [30]. Therefore, the presence of Aβ oligomers in human palatine tonsils may help to elucidate the pathogenesis of AD.

Many studies questioned the association between amyloid deposition and neuropathology in AD and investigated the potential role of pathogens [56,57,58,59]. Aβ peptides are involved in the innate immune response and protect animals from fungal and bacterial infections [60]. Recently, amyloidogenic peptide Aβ_1–42_ was shown to bind to the surface of *S. aureus* in vitro [22]. Immunocytochemistry, scanning electron microscopy, and Gram-staining analyses revealed the accelerated aggregation of Aβ_1–42_ when it was incubated with *S. aureus* [22], indicating that Aβ_1–42_ agglutination was accelerated in the presence of microorganisms. Moreover, the finding that Aβ had antimicrobial activity indicated that microbial infections induced the formation of Aβ-containing senile plaques [61]. Notably, in our samples of human tonsillar tissue, we found that there was a robust correlation between the levels of Aβ fragments (~15 kDa) and *S. aureus* in seven different tonsils (Figure 3). 

Here, we demonstrated for the first time the presence of *S. aureus* clustered around or embedded in Aβ plaques (Figure 2A). Interestingly, the confocal-microscopy images showed clustered *S. aureus* embedded in Aβ plaques in yellowish olfactory tissue similar to the tonsilloliths found in the olfactory cleft; this sample was composed of olfactory epithelium between superior turbinate and nasal septum and was collected during endoscopic endonasal skull-base surgery (Figure 2E), suggesting that Aβ peptides may be capable of ascending or descending through a cribriform plate perforated by an olfactory foramina that makes possible the passage of the olfactory nerve. The foramina in the middle of the groove allow nerves to pass to the roof of the nasal cavity; the foramina in the medial part transport nerves to the upper part of the septum; and the foramina in the lateral part transmit nerves to the superior nasal turbinate [62,63]. Several reports demonstrated the transport of Aβ peptides from the nasal cavity to the brain. In an experimental rat model, ventricle-injected Aβ peptides were observed to be transported to the nasal cavity via a nonhematogenous pathway [46]. Moreover, higher levels of Aβ peptides were detected in nasal secretions from patients with AD than in patients with other neurological diseases [47].

In the present study, we further investigated the effect of *S. aureus* on Aβ deposition in human tonsil organoids generated from human palatine-tonsil tissues. The most common bacterial isolate from human tonsillar specimens is *S. aureus* [23]. In our data, *S. aureus* was the most common bacteria in the bacterial-culture test of patients with tonsillectomy. Here, we added *S. aureus* isolated from patients to human tonsil organoids. Immunofluorescence staining showed that treatment with *S. aureus* induced approximately 3.0-fold more Aβ-positive organoids than untreated organoids, and *S. aureus* was detected around the Aβ aggregates in human tonsil organoids (Figure 4), indicating its role in Aβ-protein expression. Moreover, greater levels of Aβ were detected in the human brain organoids cultured in the presence of *S. aureus* than in the brain organoids cultured in the absence of *S. aureus* (Figure 5). Treatment with *S. aureus* resulted in approximately 40-fold more Aβ-positive cells in brain organoids than in the untreated brain organoids. These results clearly showed that *S. aureus* increased the Aβ-protein level in tonsil organoids and brain organoids, which may lead to Aβ-related AD. Interestingly, our data showed that the Aβ expression and structural disruption induced by treatment with *S. aureus* was much greater in the brain organoids than in the tonsil organoids, suggesting that infection can be fatal to the brain.

Here, we identified a pathological feature of the human palatine tonsil: a storage for AD-associated Aβ peptides as well as a bacterial reservoir. *S. aureus* was clustered around or embedded in the Aβ deposits, and some *S. aureus* and Aβ were co-localized in human tonsillar tissues as well as olfactory tissue similar to tonsilloliths found in the olfactory cleft. The patient who had olfactory tissue surgically removed was recently diagnosed with AD during a post-operative follow-up and was being treated. In addition, we evaluated the influence of pathogenic bacterial infection on Aβ-protein deposition in the inflammatory environment of human palatine-tonsil tissues. The finding that *S. aureus* increased Aβ-protein production in human tonsillar tissues suggests a possible therapeutic target in human palatine tonsils—a reservoir of Aβ protein and pathogenic bacteria. The Aβ and pathogens pooled in tonsils are thought to be related to inflammation and changes in various conditions that can induce Aβ deposition and eventually accelerate the onset of AD with age. Therefore, converting the tonsil size of a child born with tonsillitis with hypertrophy to a flat structure with the original pharyngeal mucosa via tonsillectomy may prevent the pathogen reservoir and Aβ-peptide storage. Moreover, treatment with antibiotics that kill pathogens to prevent the deposition of Aβ peptides can be used for the treatment of AD. 

## Data Availability

The datasets generated during and/or analyzed during the current study are available from the corresponding author upon reasonable request.

## Abbreviations

AD: Alzheimer’s disease; APP, amyloid precursor protein; Aβ, amyloid-β; S. aureus, Staphylococcus aureus; S. pyogenes, Streptococcus pyogenes; C. pneumoniae, Chlamydia pneumoniae; DAPI, 4′,6-diamidino-2-phenylindole; MOI, multiplicity of infection; iPSC, induced pluripotent stem cell; OSA, obstructive sleep apnea; PS1, presenilin.
